# Global longitudinal strain for detection of cardiac iron overload in patients with thalassemia: a meta-analysis of observational studies with individual-level participant data

**DOI:** 10.1186/s12947-022-00291-4

**Published:** 2022-08-12

**Authors:** Armin Attar, Alireza Hosseinpour, Hamidreza Hosseinpour, Nahid Rezaeian, Firoozeh Abtahi, Fereshte Mehdizadeh, Mozhgan Parsaee, Nehzat Akiash, Mohaddeseh Behjati, Antonella Meloni, Alessia Pepe

**Affiliations:** 1 Department of Cardiovascular Medicine, Shiraz University of Medical Sciences, Shiraz, Iran; 2 Faculty of Medicine, Shiraz University of Medical Sciences, Shiraz, Iran; 3 Echocardiography Research Center, Rajaie Cardiovascular Medical and Research Center, Iran University of Medical Sciences, Tehran, Iran; 4 Atherosclerosis Research Center, Ahvaz Jundishapur University of Medical Sciences, Ahvaz, Iran; 5 Gabriele Monasterio Foundation, Tuscan Region, Pisa, Italy

**Keywords:** Thalassemia, Iron overload, Speckle tracking, Global longitudinal strain

## Abstract

**Background:**

Although cardiac magnetic resonance (CMR) is the most reliable tool for assessment of CIO in patients with thalassemia, it is not always readily available. Recent studies have explored the potential of GLS as an alternative for diagnosis of CIO. We aimed to investigate the efficacy of global longitudinal strain (GLS) for detection of cardiac iron level (CIO).

**Methods:**

We searched SCOPUS, MEDLINE, and Embase to identify the studies which used GLS for assessment of CIO. We searched for individual participant data (IPD) in eligible studies to perform ROC curve analysis. CMR with a T2* cut-off value of 20 ms was considered as the gold standard. A meta-analysis was performed and the risk of bias was assessed using the JBI Checklist.

**Results:**

A total of 14 studies with 789 thalassemia patients (310 and 430 with and without CIO respectively and 49 with undetermined condition) were considered eligible for meta-analysis. IPDs of 405 participants were available. GLS was significantly lower in patients with CIO (-17.5 ± 2.7%) compared to those without CIO (-19.9 ± 2.3%; WMD = 1.6%, 95% CI = [0.76–2.4], *p* = 0.001, I^2^ = 77.1%) and to normal population (-20.61 ± 2.26%; WMD = 2.2%, 95% CI = [0.91–3.5], *p* = 0.001, I^2^ = 83.9%). A GLS < -19.5% could predict CIO with 92.8% sensitivity and 34.63% specificity (AUC = 0.659, 95% CI = [0.6–0.72], *p*-value < 0.0001). A GLS value < -6% has 100% positive predictive and ≥ -24.5% has 100% negative predictive values for detection of CIO.

**Conclusions:**

According to our study, GLS is a strong predictor of CIO and when CMR is not available, it may be a useful screening method for identification of CIO in thalassemia patients.

**Graphical Abstract:**

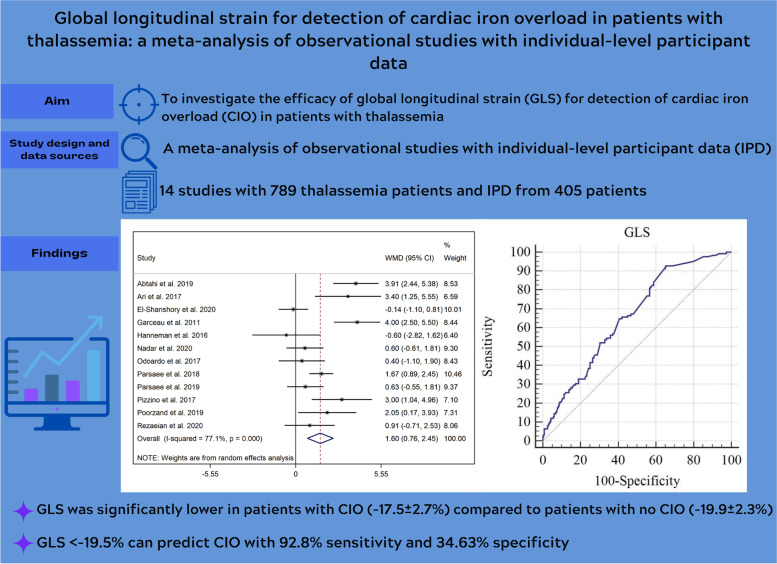

**Supplementary Information:**

The online version contains supplementary material available at 10.1186/s12947-022-00291-4.

## Background

Cardiomyopathy induced by iron deposition in patients with thalassemia is a reversible phenomenon when iron chelation therapy is started on time. Therefore, early detection of cardiac iron overload (CIO) in the prevention of progressive heart failure is vital [1, 2]. Cardiac magnetic resonance (CMR) imaging has been proposed as the most reliable and sensitive non-invasive tool to evaluate the iron level of myocardium [3–5]. Values of T2* less than 20 and 10 ms are indicative of CIO and severe CIO, respectively. A study has estimated the prevalence of CIO in magnetic resonance imaging (MRI) to be 25% [6]. Cardiac T2* values have a prognostic role, allowing the identification of thalassemia patients at risk for cardiac complications and needing an intensification of chelation therapy [7]. However, CMR for iron overload screening in patients with thalassemia has the disadvantages of limited availability and high costs [8]. This emphasizes the importance of finding alternative tools to investigate the state of cardiac iron overload.

Previous studies have shown the benefits of novel echocardiographic techniques such as strain imaging and tissue imaging [9, 10] for detection of early and subclinical myocardial dysfunction syndromes. Speckle-tracking echocardiography (STE) is a novel imaging technique for evaluating the myocardial function and diagnosing the deformation of myocardium using exclusive parameters including three strain components- longitudinal, circumferential, and radial [9, 10]. Recently, STE has been used for early detection of ventricular dysfunction in patients with transfusion dependent thalassemia (TDT) [11]. Longitudinal strain was found to be significantly different among patients with β-thalassemia major and healthy subjects in several studies [12–14]. Furthermore, global longitudinal strain (GLS) has been proposed as a novel predictor of CIO in patients with TDT and hence, a promising alternative for cardiac T2*. However, most of the studies have only done a correlation assessment between GLS and CMR T2* values. Nevertheless, a more important question is that how these echocardiographic findings differ in patients with a cardiac MRI T2* values less than 20 ms, as an indicator of myocardial iron deposition. A few studies with limited sample size have performed ROC curve analysis to find appropriate cut off values and have suggested different GLS cut-off values for detection of cardiovascular involvement [15–17]. Therefore, in this study we aimed to find a more precise GLS cut-off value using individual participant data analysis. Also, available echocardiographic parameters such as GLS and left ventricular ejection fraction (LVEF) were analyzed to see if there is any correlation between CIO and echocardiographic markers.

## Methods

### Protocol

This study was registered with PROSPERO (registration number: CRD42021230361) and conducted according to the recommendations provided by Preferred Reporting Items for a Systematic Review and Meta-Analysis of Individual Participant Data (PRISMA-IPD) statement 2015 [18].

### Eligibility criteria

Eligible studies were all the original observational articles which compared the parameters of two-dimensional and three-dimensional speckle tracking echocardiography including GLS and LVEF between subgroups of patients with β-thalassemia major based on their cardiac T2* values (T2* ≥ 20 ms identifying patients with no CIO and T2* < 20 ms identifying thalassemia patients with CIO). The studies which only compared the CMR imaging parameters or echocardiographic data between patients with thalassemia and healthy subjects were excluded from the present meta-analysis since they did not divide thalassemia patients based on their CIO status.

### Search strategy

**Fig. 1:**
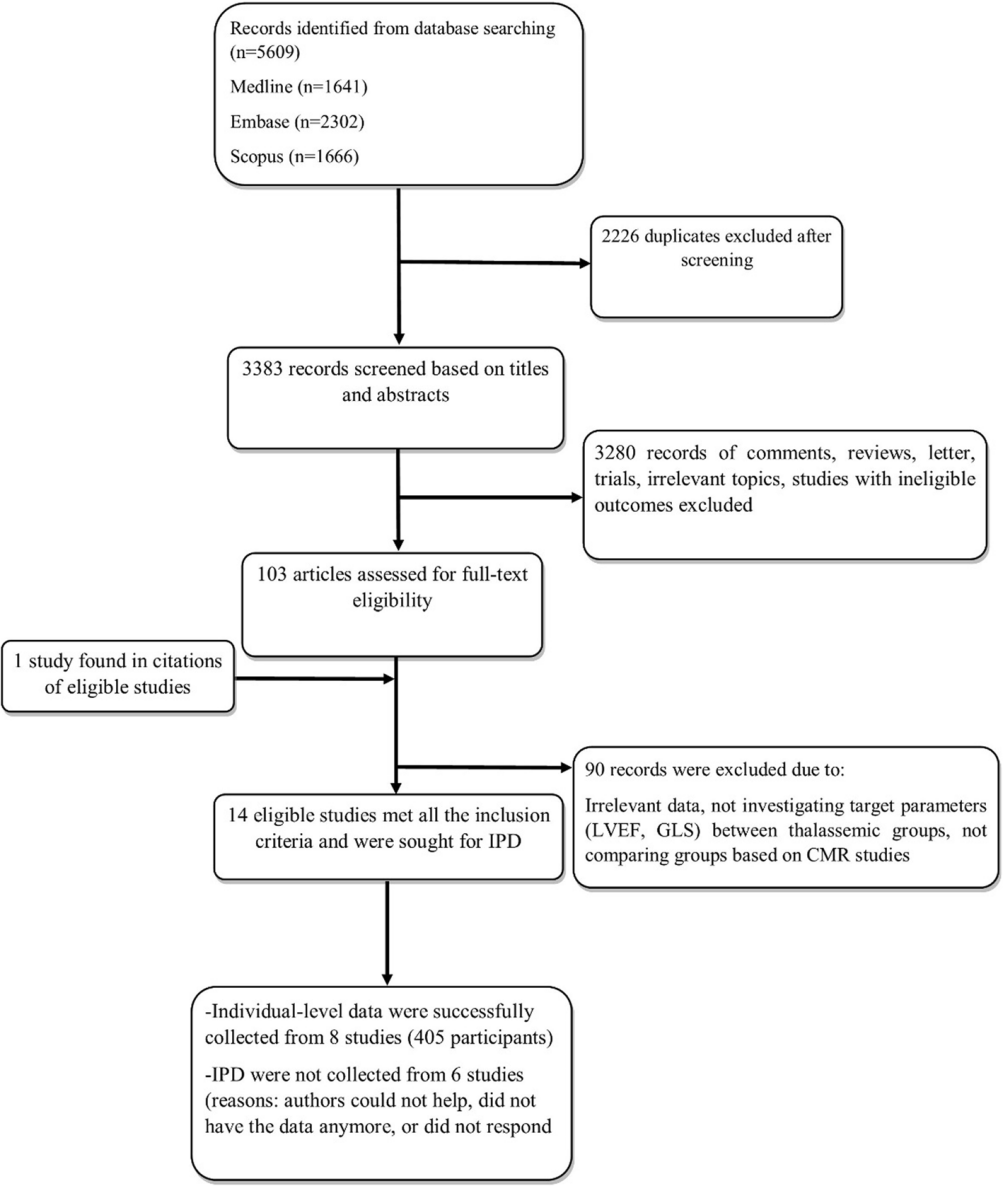
PRISMA Flowchart for selection of the eligible studies

We performed a categorized and comprehensive search in electronic databases including SCOPUS, MEDLINE and Embase. The primary search was run on 10^th^ of January 2020 and it was updated on 30^th^ of October 2021. No specific restriction was applied to our search and the sample size. All the articles with no time limits were included. Only the studies in the English language with available full-text were included as eligible. Additional relevant studies were identified through the references list of the included articles. The following keywords were searched: (Thalassemia) AND ((cardiomyopathy) OR (cardiac iron overload) OR (echocardiography) OR (cardiac MRI) OR (global longitudinal strain) OR (speckle-tracking). The related PRISMA flow diagram is illustrated in Fig. 1.

### Study selection

Two investigators (AA, AH) screened the titles and abstracts for relevant studies. After removing the duplicates, studies that did not meet the selection criteria through reviewing titles or/and abstracts were excluded. For potential relevant studies and for articles with unclear eligibility, the full text was reviewed. Any conflicts were resolved by discussion between the two reviewers (AA, AH); in case of further disagreements, a third party would decide.

### Data collection

Individual participant data (IPD) of eligible studies were requested to be included in our meta-analysis. We contacted the corresponding authors via email and explained the study goals and asked for their raw data on the participants’ values (GLS and T2*) for generating a ROC curve and providing cut-off value with sensitivity and specificity as well as baseline characteristics including age and sex. A reminder email was sent to all the corresponding authors 2 weeks after the initial email if they did not respond. The received data were merged into one dataset and one of the authors (AA) explored the received data integrity by doing internal consistency checks. Also, for additional analyses, sample size as well as all available echocardiographic parameters were gathered by one of the reviewers (AH) from both groups with thalassemia (T2* ≥ 20 and T2* < 20) and also in the control groups if available.

### Risk of bias assessment

We assessed the quality of the selected studies using the JBI Checklist for Diagnostic Test Accuracy Studies which comprises 10 questions [19, 20]. The possible answers to the questions include *Yes*, *No*, *Unclear* or *Not Applicable*. Two investigators (AA, AH) checked the risk of bias using the mentioned tool.

### Data synthesis and statistical analysis

A one-stage individual participant data meta-analysis was performed using the data collected from the available studies on GLS to create a receiver-operating characteristic (ROC) curve. We used Youden index to calculate an optimal threshold (cut-off) in addition to its sensitivity and specificity. Also, area under the curve (AUC), standard error (SE), and 95% confidence interval (CI) were reported for the results.

Additional analyses were also conducted. Values of echocardiographic markers including GLS, left ventricular ejection fraction (LVEF), global circumferential strain (GCS), global radial strain (GRS), E/A, E/E’, and deceleration time between patients with thalassemia with and without evident cardiac iron overload based on their CMR T2^*^ were pooled for generating forest plots and calculating the weighted mean difference (WMD) and 95% CI with DerSimonian and Laird method. Another analysis was made to compare the markers GLS and LVEF between the healthy subjects and thalassemia patients as well. The magnitude of heterogeneity was estimated using I^2^ and values of 25%, 50%, and 75% were considered low, moderate, and high degree of inconsistency, respectively. Random effects model was used for all the analyses. For possible publication bias, Egger’s test and related funnel plots were evaluated. A *p*-value of < 0.05 was considered significant for all the analyses. All the statistical analyses were conducted using Stata software version 14.2 (StataCorp LP, College Station, TX, USA) and MedCalc program version 19.6.1 (MedCalc Software Ltd, Ostend, Belgium).

## Results

### Study selection and included studies characteristics

After an initial search through the databases, 5609 records were identified for screening. Following the removal of duplicates, titles and abstracts of the remaining studies were investigated and irrelevant or ineligible records were eliminated. Finally, 14 studies with 789 thalassemia participants (310 and 430 with and without CIO respectively and 49 with undetermined condition) met all the eligibility criteria for the systematic review and meta-analysis [15–17, 21–31] (Table 1 summarized their characteristics) (Fig. 1). Eight studies with 405 patients with β-thalassemia accepted to share their data to be analyzed for IPD [12, 15, 17, 21, 22, 24, 30, 31]. It is important to note that some authors have provided us with data from more number of patients than those used in their original investigation.

**Table 1:** Characteristics of the 14 eligible studies for meta-analysis

Studies	Year of publication	Country	Age	Male (%)	No. of patients		Echocardiographic data			
					T2* ≥ 20	T2* < 20	**GLS**		**LVEF**	
							T2* ≥ 20	T2* < 20	T2* ≥ 20	T2* < 20
Pizzino et al. [21]	2017	Italy	37.4 ± 10	53%	22	6	21.3 ± 2.7	18.3 ± 2	60.2 ± 4.8	58 ± 2.4
Parsaee et al. [15]	2018	Iran	30.79 ± 9.37	45%	81	41	-19 (-18 to -20)	-17 (-19 to -16)	63 (59–68)	58 (53–63)
Abtahi et al. [22]	2019	Iran	23.7 ± 5	56%	28	22	21.55 ± 2.68	17.64 ± 2.56	58.5 ± 5	59.32 ± 4.9
Ari et al. [16]	2017	Turkey	13.3 ± 16.1	50%	20	10	-23.10 ± 2.20	-19.70 ± 3.10	68.3 ± 3.5	
Rezaeian et al. [17]	2020	Iran	24.66 ± 6.23	57.1%	25	66	-13.81 ± 3.25	-12.9 ± 4.18	52.0 ± 12.4	
Hanneman et al. [23]	2016	Canada	34.6 ± 9.5	53%	11	19	-18.8 ± 3.5	-19.4 ± 1.8	58.5 ± 5.5	60.4 ± 4.4
Parsaee et al. [24]	2019	Iran	31.96 ± 7.5	44.4%	25	20	-22.36 ± 2.34	-21.73 ± 1.71	-	
Garceau et al. [25]	2011	Canada	34.4 ± 11	46.6%	23	22	-20 ± 2	-16 ± 3	64 ± 5	59 ± 7
Nadar et al. [26]	2020	Oman	26.3 ± 6.1	42.8%	61	23	-19.2 ± 2.8	-18.6 ± 2.4	59.5 ± 6.7	60.4 ± 5.2
El-Shanshory et al. [4]	2020	Egypt	10.9 ± 3.7	66.6%	68	32	-21.23 ± 2.68	-21.375 ± 2.06	54.88 ± 12.47	49.88 ± 14.04
Djer et al. [28]	2020	Indonesia	13–41	50%	16	14	-		65.6 (60.6–75.5)	64.8 (60.5–75)
Barzin et al. [29]	2012	Iran	23.5 (15–37)	48.4%	16	14	-		60.3 ± 3.99	51.1 ± 4.39
Poorzand et al. [30]	2019	Iran	23.51 ± 6.2	52.35%	30	14	-20.28 ± 1.67	-18.23 ± 3.41	58.6 ± 2.87	56.05 ± 8.8
Odoardo et al. [31]	2017	Italy	36.6 ± 7	38.2%	34	21	-19.2 ± 3	-18.8 ± 2.6	61 ± 5.3	60.6 ± 6.2

### Global longitudinal strain

**Fig. 2:**
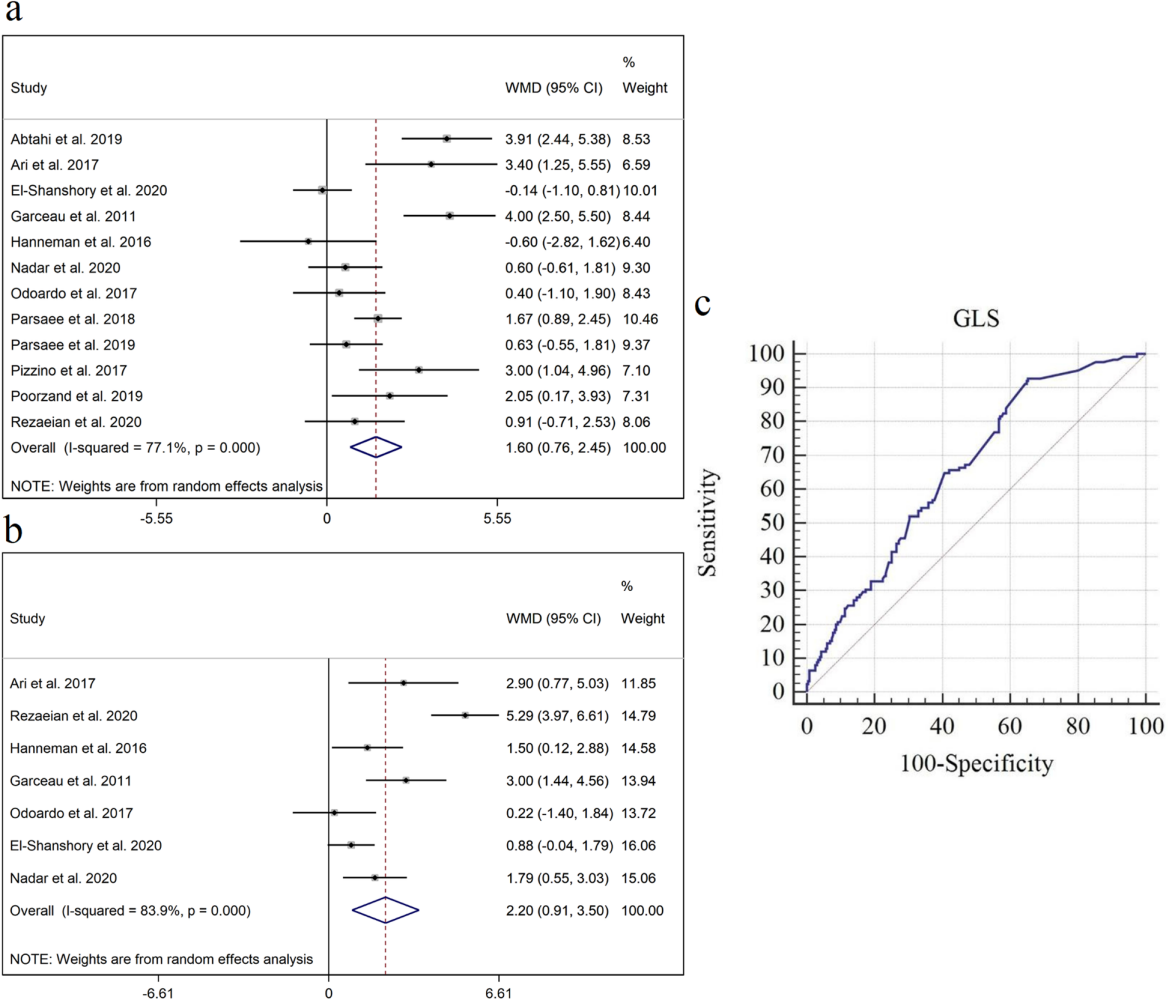
Forest plot of comparison of GLS between **a** patients with β-thalassemia with CIO and without any evident CIO and **b** control group and patients with thalassemia **c **ROC curve analysis of GLS (a cut-off value of -19.5 could be employed for early detection of myocardial iron overload in patients with β-thalassemia)

The pooled mean ± standard deviation (SD) GLS was -17.48 ± 2.69 and -19.88 ± 2.33, respectively in thalassemic patients with and without CIO. Comparison of GLS between the thalassemic groups with and without CIO showed that GLS was significantly lower in patients with CIO compared to those without CIO (WMD = 1.60, 95% CI = [0.76—2.45], *p* = 0.001, I^2^ = 77.1%) (Fig. 2a). Heterogeneity decreased from 77.1% to 52.3% after subtraction of outliers although excluding the outliers diminished the level of association (WMD = 0.90, 95% CI = [0.25—1.55], *p* = 0.007). Also the mean ± SD GLS in the control group was -20.61 ± 2.26 and the difference between the control and thalassemia groups with overload was also found to be significant (WMD = 2.20, 95% CI = [0.91—3.50], *p* = 0.001, I^2^ = 83.90%) (Fig. 2b). After excluding the outliers the level of heterogeneity decreased from 83.9% to 48% but the level of association remained high (WMD = 1.58, 95% CI = [0.79—2.37], *p* = 0.001).

Meta-analysis of the IPD showed a cardiac iron overload prevalence of 35.1%. Characteristic ROC curve analysis revealed that an optimal cut-off value of -19.5 for GLS could predict the cardiac iron overload in patients with thalassemia with 92.8% sensitivity and 34.63% specificity (AUC = 0.659, SE = 0.0291, 95% CI = [0.60—0.72], *p*-value < 0.0001) (Fig. 2c). A GLS value < -6 had 100% positive predictive and ≥ -24.5 had 100% negative predictive value for detection of CIO.

### Left ventricular ejection fraction

**Fig. 3:**
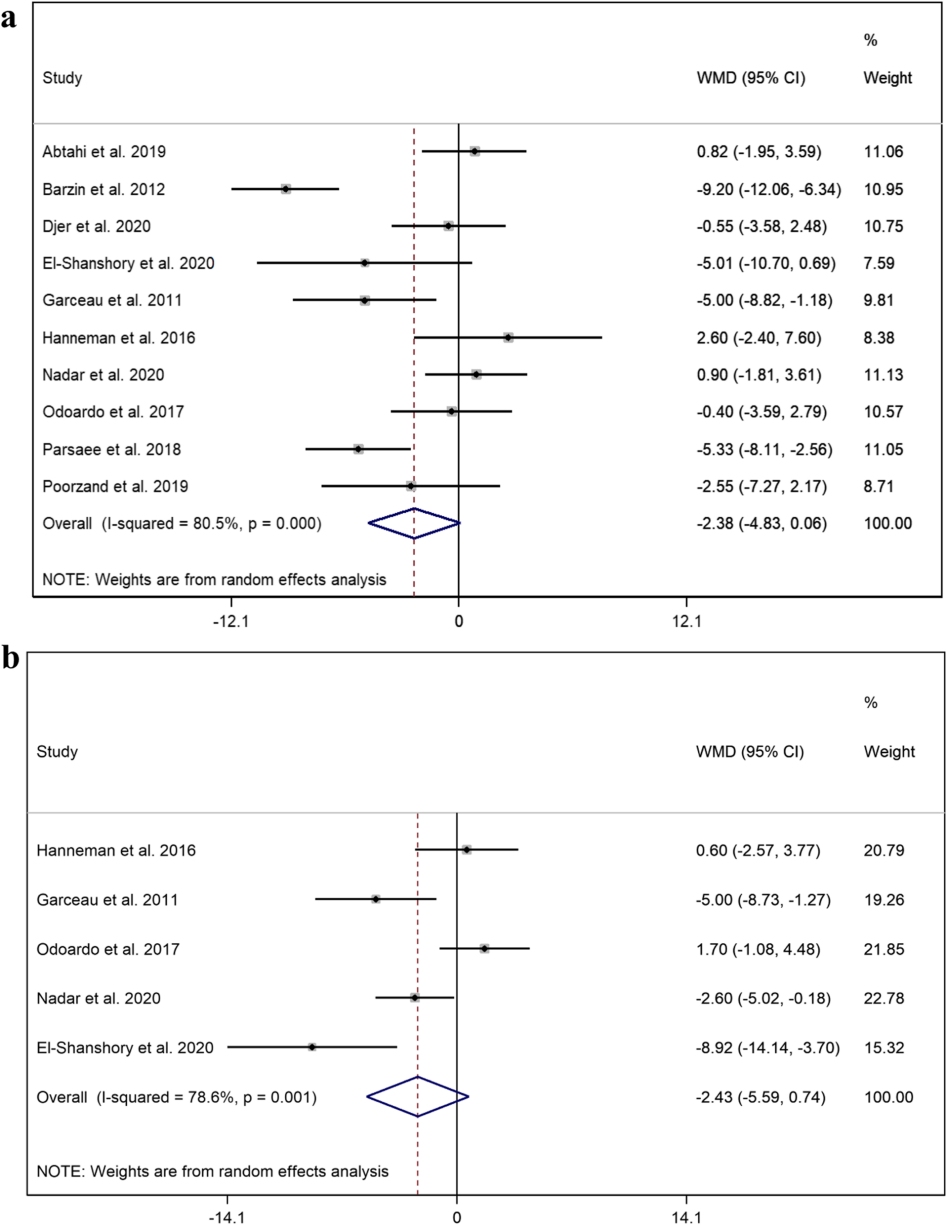
Forest plot of the comparison of LVEF between **a **thalassemic participants with and without CIO and **b **healthy subjects and thalassemic group

Pooled mean ± SD of LVEF in the thalassemic groups with and without CIO and also control group was 57.74 ± 6.92, 60.12 ± 6.84, and 62.38 ± 4.13, respectively. No significant differences were observed regarding LVEF in patients with thalassemia with and without CIO (WMD = -2.38, 95% CI = [-4.83—0.06], *p* = 0.056, I^2^ = 80.5%) (Fig. 3a) Subtracting outliers reduced the level of heterogeneity from high to low (I^2^ = 0.0%) (WMD = 0.22, 95% CI = [-1.12—1.55], *p* = 0.752). Also between healthy subjects and thalassemia groups with CIO (WMD = -2.42, 95% CI = [-5.59—0.74], *p* = 0.133, I^2^ = 78.6%) (Fig. 3b). The level of heterogeneity remained high after removing outliers (I^2^ = 71.9%).

### Other echocardiographic parameters

**Fig. 4:**
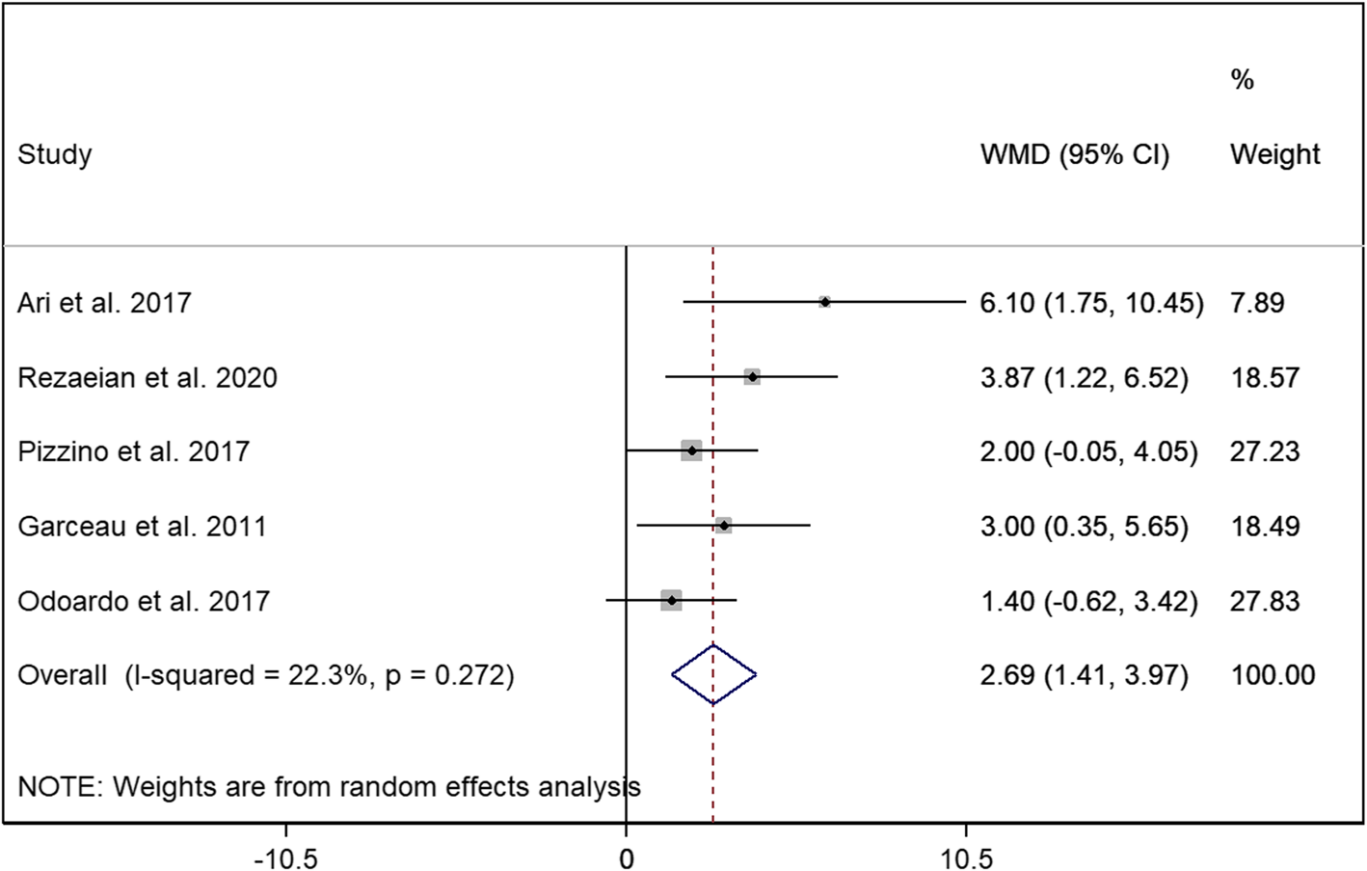
Forest plot of comparing GCS between patients with thalassemia with and without CIO

There was a significant correlation between lower levels of GCS and myocardial iron overload (WMD = 2.69, 95% CI = [1.41—3.97], *p* < 0.001, I^2^ = 22.3%) with possible publication bias according to Egger’s test (*p* = 0.016) although evaluation of trim-and-fill method showed minimal evidence for publication bias (Fig. 4). In contrast, no difference was found regarding the values of GRS between the two groups of thalassemia (WMD = -3.853, 95% CI = -7.796 to 0.091, *p* = 0.055, I^2^ = 0.00%) (Supplementary material, Figure-S1). E/A ratio and E/E’ ratio comparison did not reach a significant level between the two groups (E/A: WMD = 0.159, 95% CI = -0.104 to 0.422, *p* = 0.237, I^2^ = 84.4%) (Supplementary material, Figure-S2) (E/E’: WMD = 1.006, 95% CI = -1.574 to 3.585, *p* = 0.445, I^2^ = 95.6%) (Supplementary material, Figure-S3). No correlation was also observed regarding the deceleration time between the two thalassemia groups (WMD = -5.343, 95% CI = -38.906 to 28.221, *p* = 0.755, I^2^ = 92.0%) (Supplementary material, Figure-S4).

### Risk of bias of the included studies

After evaluating the quality of the eligible studies using JBI Checklist, we decided to include all the studies in the meta-analysis due to their high-quality. Table 2. summarizes the risk of bias assessment of the eligible studies.

**Table 2:** Quality assessment of the included studies using JBI Checklist for Diagnostic Test Accuracy Study

Studies	(1)Random sampling	(2)Avoidance of case control design	(3)Avoidance of inappropriate exclusions	(4)Blinded interpretation of index test results	(5)Use of pre-specified threshold	(6)Reference standard correctly classifies the target condition	(7)Blinded interpretation of reference standard	(8)Appropriate interval between index test and reference standard	(9)Patients receiving the same reference standard	(10)All the patients including in the analysis
Parsaee et al. [15]	No	No	Yes	Unclear	Yes	Yes	Yes	Yes	Yes	Yes
Ari et al. [16]	No	No	Yes	Unclear	Yes	Yes	Yes	Yes	Yes	Yes
Rezaeian et al. [17]	Yes	No	Yes	Unclear	Yes	Yes	Yes	Yes	Yes	Yes
Pizzino et al. [21]	No	No	Yes	Unclear	Yes	Yes	Yes	Yes	Yes	Yes
Hanneman et al. [23]	Yes	No	Yes	Unclear	Yes	Yes	Yes	Unclear	Yes	Yes
Parsaee et al. [24]	No	No	Yes	Unclear	Yes	Yes	Yes	Yes	Yes	Yes
Garceau et al. [25]	No	No	Yes	Unclear	Yes	Yes	Yes	Yes	Yes	Yes
Abtahi et al. [22]	Yes	No	Yes	Unclear	Yes	Yes	Yes	Yes	Yes	Yes
Nadar et al. [26]	Yes	No	Yes	Unclear	Yes	Yes	Yes	Yes	Yes	Yes
El-Shanshory et al. [4]	Yes	No	Yes	Unclear	Yes	Yes	Yes	Yes	Yes	Yes
Djer et al. [28]	Yes	No	Yes	Unclear	Yes	Yes	Yes	Yes	Yes	Yes
Barzin et al. [29]	No	No	Yes	Unclear	Yes	Yes	Yes	Yes	Yes	Yes
Poorzand et al. [30]	Yes	No	Yes	Unclear	Yes	Yes	Yes	Yes	Yes	Yes
Odoardo et al. [31]	No	No	Yes	Unclear	Yes	Yes	Yes	Yes	Yes	Yes

Evaluation of the funnel plots for the main outcomes (GLS and LVEF) demonstrated the presence of asymmetrical distribution among the studies (Supplementary material, Figure-S5, 6, 7, 8). However, *p*-values of Egger’s test did not reach a significant level for publication bias (*p* = 0.144 for GLS between thalassemia patients with and without CIO, *p* = 0.510 for GLS between thalassemia and control group, *p* = 0.777 for LVEF between thalassemia patients with and without CIO, *p* = 0.263 for LVEF between the thalassemia and control group). For sensitivity analysis, we removed each single study from all the analyses to see their impact on the summary of results and no significant change was observed for all the parameters.

## Discussion

Here for the first time, we performed a meta-analysis to evaluate the efficacy of speckle tracking echocardiography for detection of CIO using IPD and found that GLS might be a promising tool for detection of this pathology. We used a T*2 CMR value of 20 ms as the gold standard and found that GLS was significantly lower in patients with CIO (-17.5 ± 2.7) compared to those without CIO (-19.9 ± 2.3) and to normal population (-20.6 ± 2.3). According to IPD analysis, a GLS < -19.5 could predict CIO with a sensitivity of 92.8% and specificity of 34.63%. A GLS value < -6 had 100% positive predictive and ≥ -24.5 had 100% negative predictive value for detection of CIO.

Although CMR imaging is the most accurate non-invasive technique for diagnosis of myocardial iron overload, it has some limitations and disadvantages. Cardiac MRI is a time-consuming and costly technique and it is not the most accessible option compared to other diagnostic techniques including echocardiography in all regions of the world [8]. Several studies have evaluated the correlation between echocardiographic parameters and CMR findings. A study conducted by Aypar et al. on septal systolic myocardial (S_m_) velocity and septal early diastolic myocardial (E_m_) velocity showed a significant relationship with T_2_^*^ [32]. In another study by Magri et al. [33], systolic strain of right ventricular free wall, systolic strain of the septal wall, and systolic strain of lateral wall were significantly associated with T_2_^*^. However, despite the importance of the mentioned findings, the most important correlations to investigate are those with cardiac MRI T_2_^*^ values ​​less than 20 ms as an indicator of myocardial iron overload. Pizzino and colleagues found that GLS showed a significant correlation with T2* values (*R* =  − 0.49; *P* = 0.001), and it was significantly lower in patients with a T2* value lower than 20 ms (− 18.3 ± 2 vs. − 21.3 ± 2.7) [21]. Garceau and colleagues found a strong and direct logarithmic relationship between GLS and T_2_^* ^[25]. Similar findings were noted in other studies [12, 15, 22]. On the contrary, some other studies did not find such correlation between CMR T2^*^ and GLS [23, 24, 26]. Our analysis showed that GLS was significantly lower in patients with iron deposition than those without it and normal healthy population. However, it is very important to find a cut off value based on which diagnosis or exclusion of CIO can be done.

**Fig. 5:**
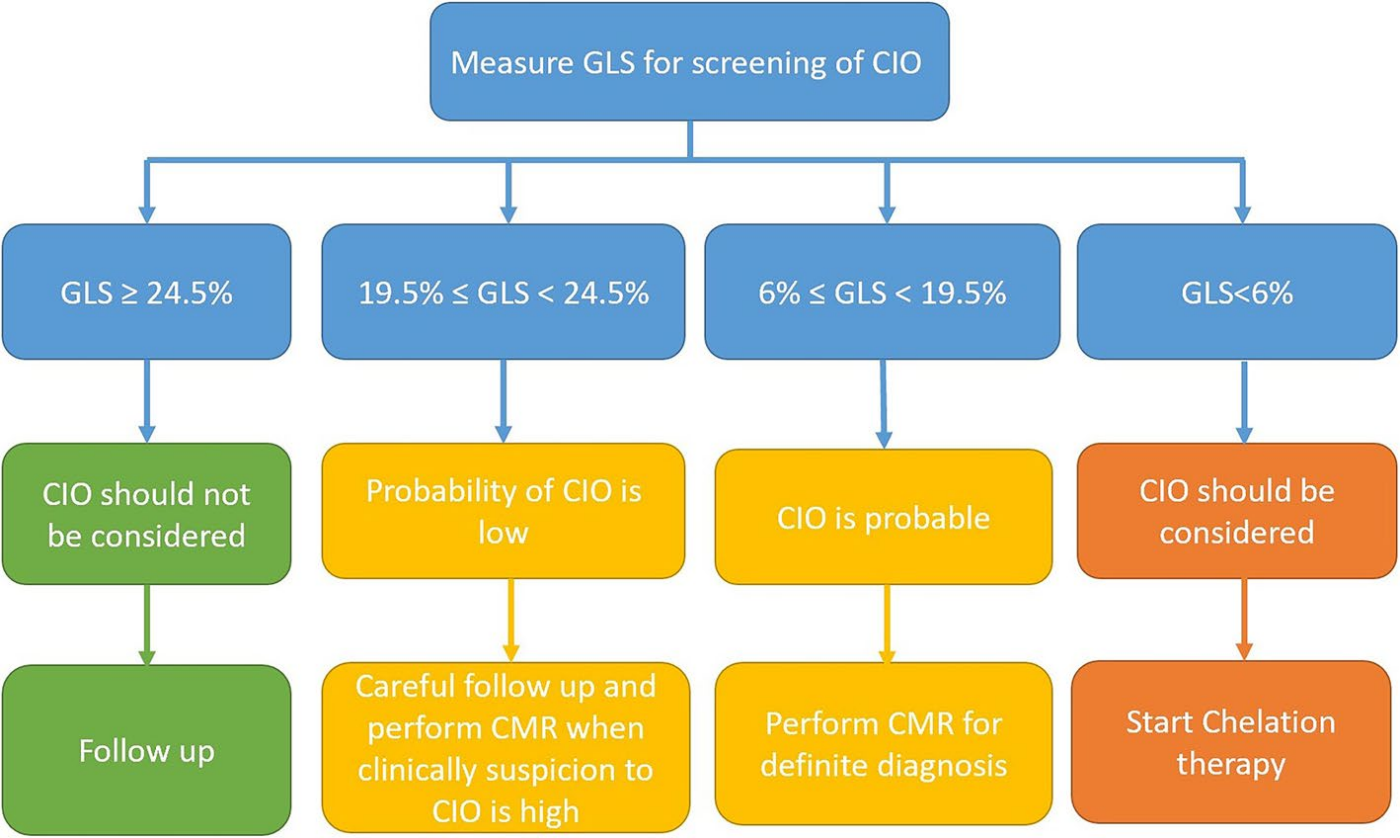
Proposed algorithm for using GLS measurement to screen cardiac Iron overload (CIO). Note that for better understanding, the GLS values are mentioned in their absolute numerical form

In the present study, we sought to establish a cut-off value using IPD meta-analysis for STE markers to help diagnose CIO in thalassemia patients. Our analysis revealed that A GLS value less than 6% was diagnostic for CIO, and ≥ -24.5% could reliably rule out the presence of CIO. Also, a threshold value of less than -19.5% with a 92.8% sensitivity and 34.63% specificity can be used for screening purposes due to its high sensitivity. It is noteworthy that although this cut-off value represents a high sensitivity for detection of CIO, it should be practiced with caution since it has a low level of specificity. Only a few studies have performed a ROC curve analysis to find a cut off value. Pizzino and colleagues showed that patients with impaired GLS (< − 19.5%) had a significant higher risk of showing significant cardiac iron deposition (Odds ratio = 17) [21]. Similarly, Abtahi et al. [22] found that a GLS threshold of 19.5, as the cut off value, could detect Iron deposition with a sensitivity of 82.14% and specificity of 86.36%. Based on the findings from this study, an algorithm (Fig. 5) may be suggested to explain how to use speckle tracking echocardiography and GLS measurement for screening and detection of myocardial iron overload.

This study had some limitations. First, we could not find IPD from all the studies. Furthermore, it is known that different echocardiography machines use various software and may produce unequal final GLS results and since we have performed a met-analysis this issue could not be addressed. In addition, all studies were cross-sectional and we were unable to show whether early detection of cardiac iron overload with GLS can be translated into a higher risk of heart failure in the future.

## Conclusions

Recent studies have explored the potential of STE markers for early detection of CIO in individuals with β-thalassemia to prevent iron deposition induced heart failure. Among all the candidates, in this meta-analysis we showed that GLS was the most favorable echocardiographic marker in diagnosing CIO. Furthermore, we provided a ROC curve and reliable cut-off value for GLS that can be employed for detection of CIO in patients with β-thalassemia using IPD meta-analysis.

## Supplementary Information

**Additional file 1: Figure-S1. **Forest plot of comparison of GRS between thalassemia groups with and without CIO. 

**Additional file 2:** **Figure-S2.** Forest plot of comparison of E/A between thalassemia groups with and without CIO.

**Additional file 3: Figure-S3.** Forest plot of comparison of E/E’ between thalassemia groups with and without CIO.  

**Additional file 4: Figure-S4. **Forest plot demonstrating comparison of deceleration time between thalassemia groups with and without CIO.

**Additional file 5: Figure-S5.** Funnel plot of GLS between thalassemic groups.

**Additional file 6. Figure-S6.** Funnel plot of GLS between thalassemic groups with CIO and healthy participants.

**Additional file 7: Figure-S7.** Funnel plot of LVEF between thalassemic groups.

**Additional file 8: Figure-S8. **Funnel plot of LVEF between thalassemic groups with CIO and healthy participants.

## Data Availability

The data underlying this article will be shared on reasonable request to the corresponding author.

## Abbreviations

CIO: Cardiac iron level
CMR: Cardiac magnetic resonance
GLS: Global longitudinal strain
IPD: Individual participant data
MRI: Magnetic resonance imaging
EF: Ejection fraction
FS: Fractional shortening
STE: Speckle-tracking echocardiography
GCS: Global circumferential strain
GRS: Global radial strain
ROC: Receiver-operating characteristic
WMD: Weighted mean difference
AUC: Area under the curve
SE: Standard error
CI: Confidence interval
LVEF: Left ventricular ejection fraction
GRS: Global radial strain
TDT: Transfusion dependent thalassemia
