# Supplementary figures and images for: Global longitudinal strain for detection of cardiac iron overload in patients with thalassemia: a meta-analysis of observational studies with individual-level participant data
Source: Cardiovasc Ultrasound. 2022 Aug 12;20:22. doi: 10.1186/s12947-022-00291-4 (PMC9373500; doi:10.1186/s12947-022-00291-4)

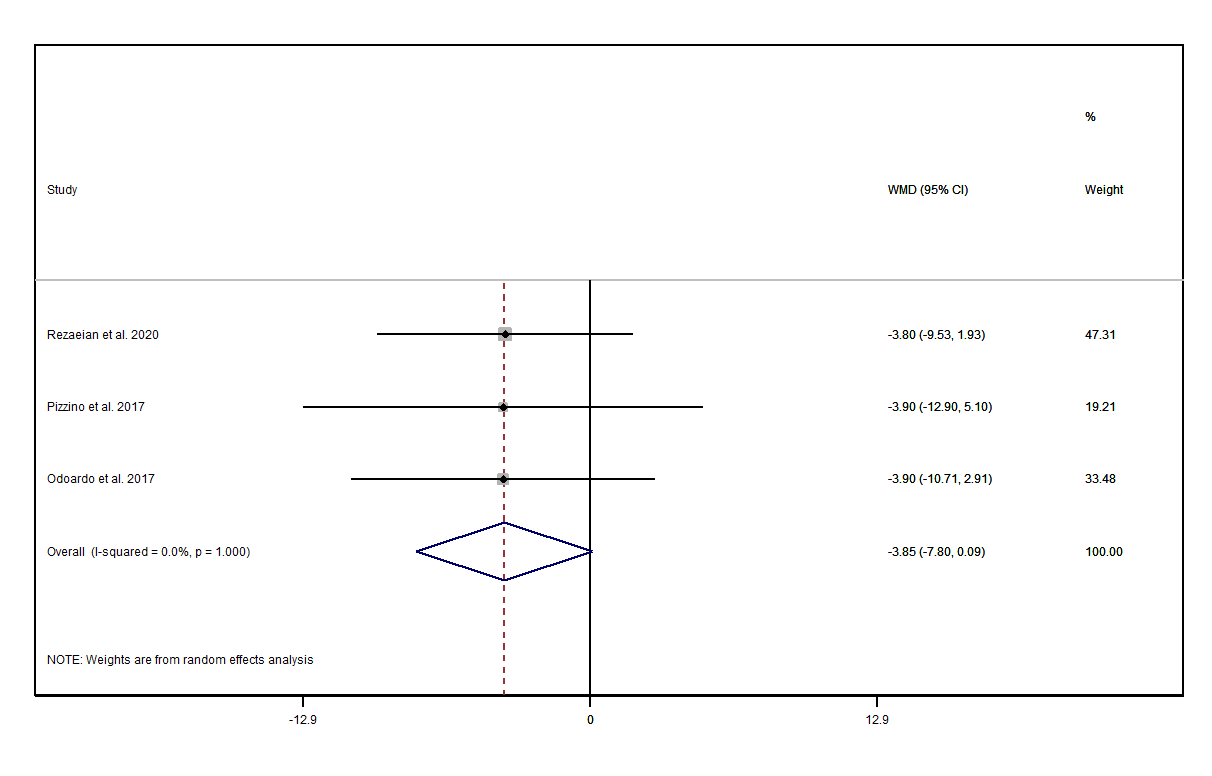

Supplement: Supplementary file 1 — Additional file 1: Figure-S1. Forest plot of comparison of GRS between thalassemia groups with and without CIO. [file 12947_2022_291_MOESM1_ESM.tif]

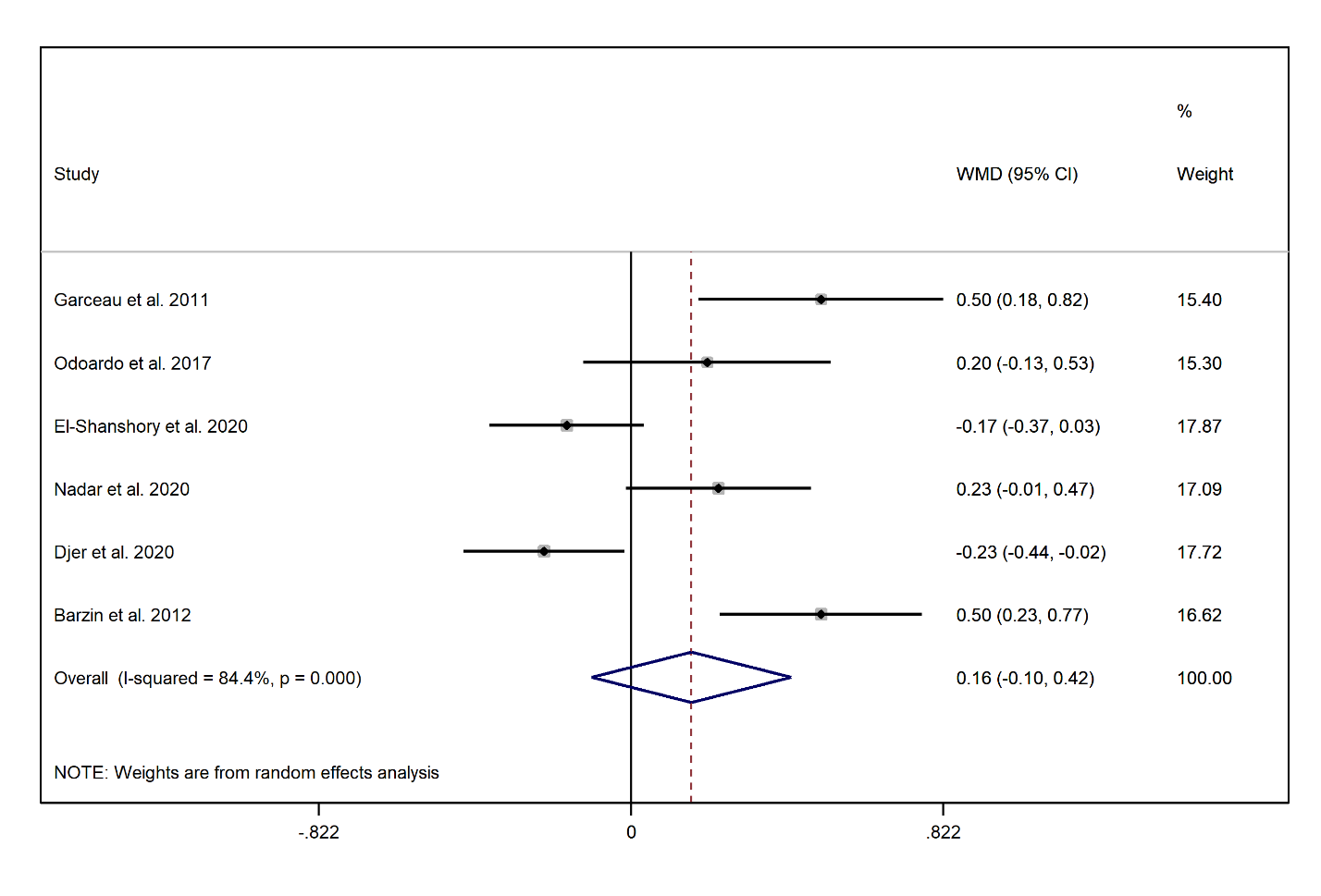

Supplement: Supplementary file 2 — Additional file 2: Figure-S2. Forest plot of comparison of E/A between thalassemia groups with and without CIO. [file 12947_2022_291_MOESM2_ESM.tif]

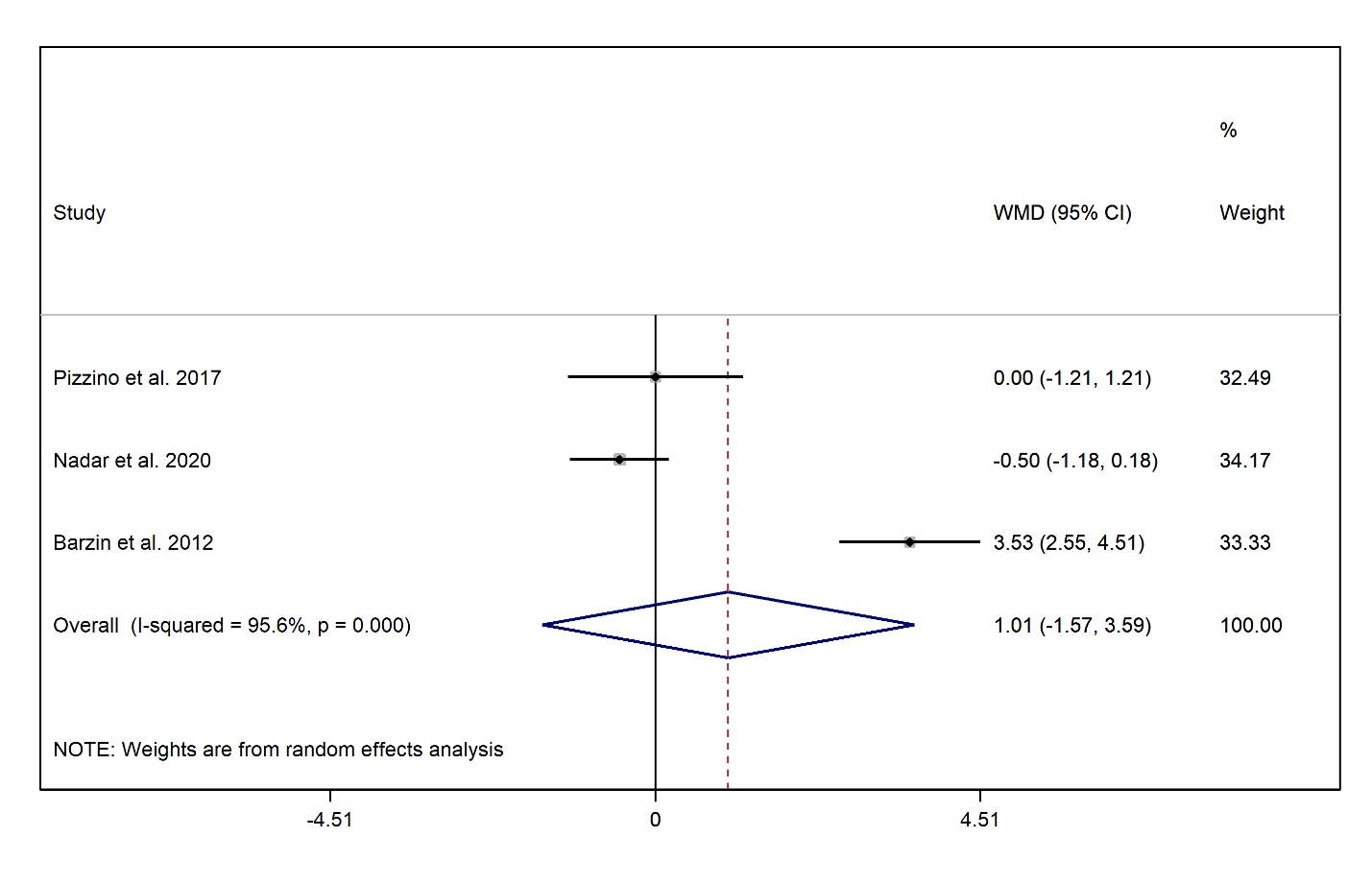

Supplement: Supplementary file 3 — Additional file 3: Figure-S3. Forest plot of comparison of E/E’ between thalassemia groups with and without CIO. [file 12947_2022_291_MOESM3_ESM.tif]

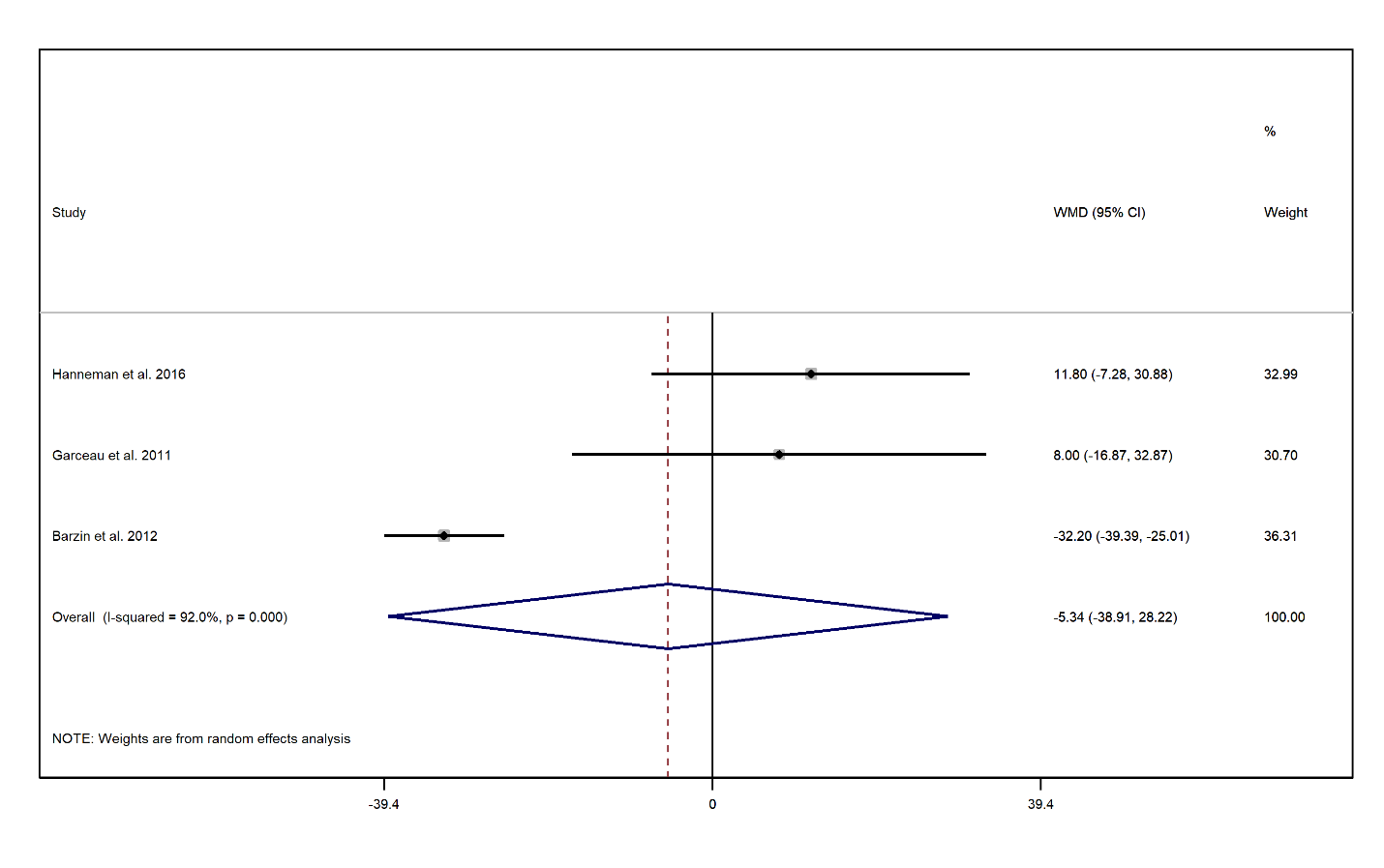

Supplement: Supplementary file 4 — Additional file 4: Figure-S4. Forest plot demonstrating comparison of deceleration time between thalassemia groups with and without CIO. [file 12947_2022_291_MOESM4_ESM.tif]

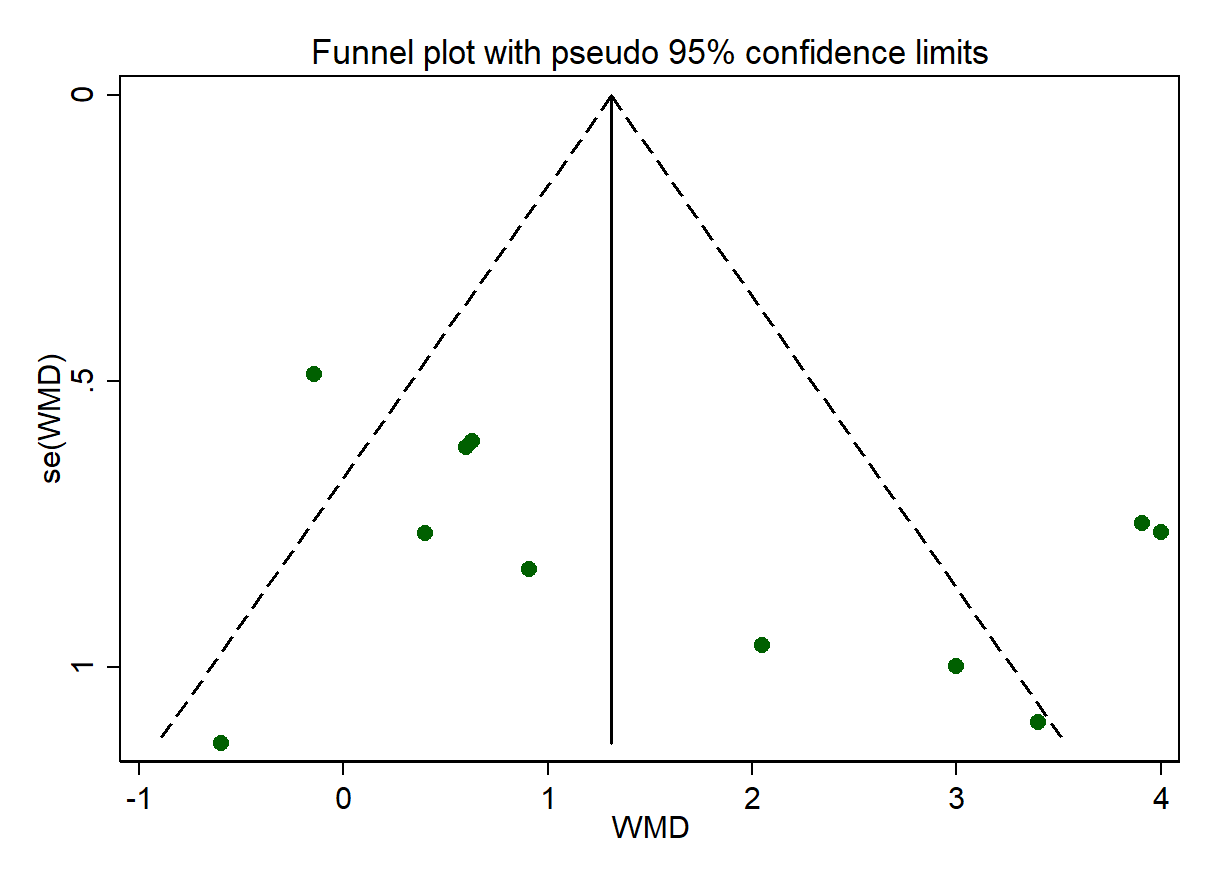

Supplement: Supplementary file 5 — Additional file 5: Figure-S5. Funnel plot of GLS between thalassemic groups. [file 12947_2022_291_MOESM5_ESM.tif]

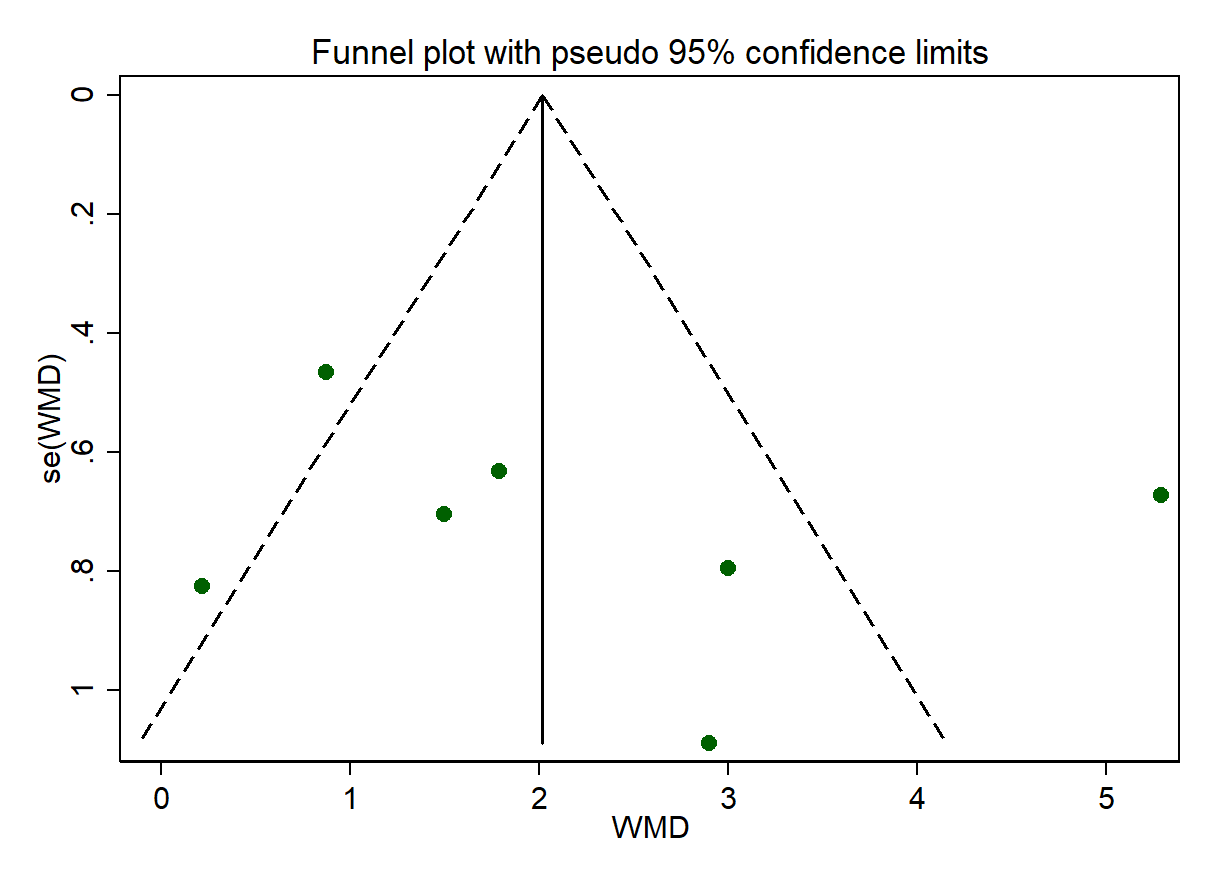

Supplement: Supplementary file 6 — Additional file 6. Figure-S6. Funnel plot of GLS between thalassemic groups with CIO and healthy participants. [file 12947_2022_291_MOESM6_ESM.tif]

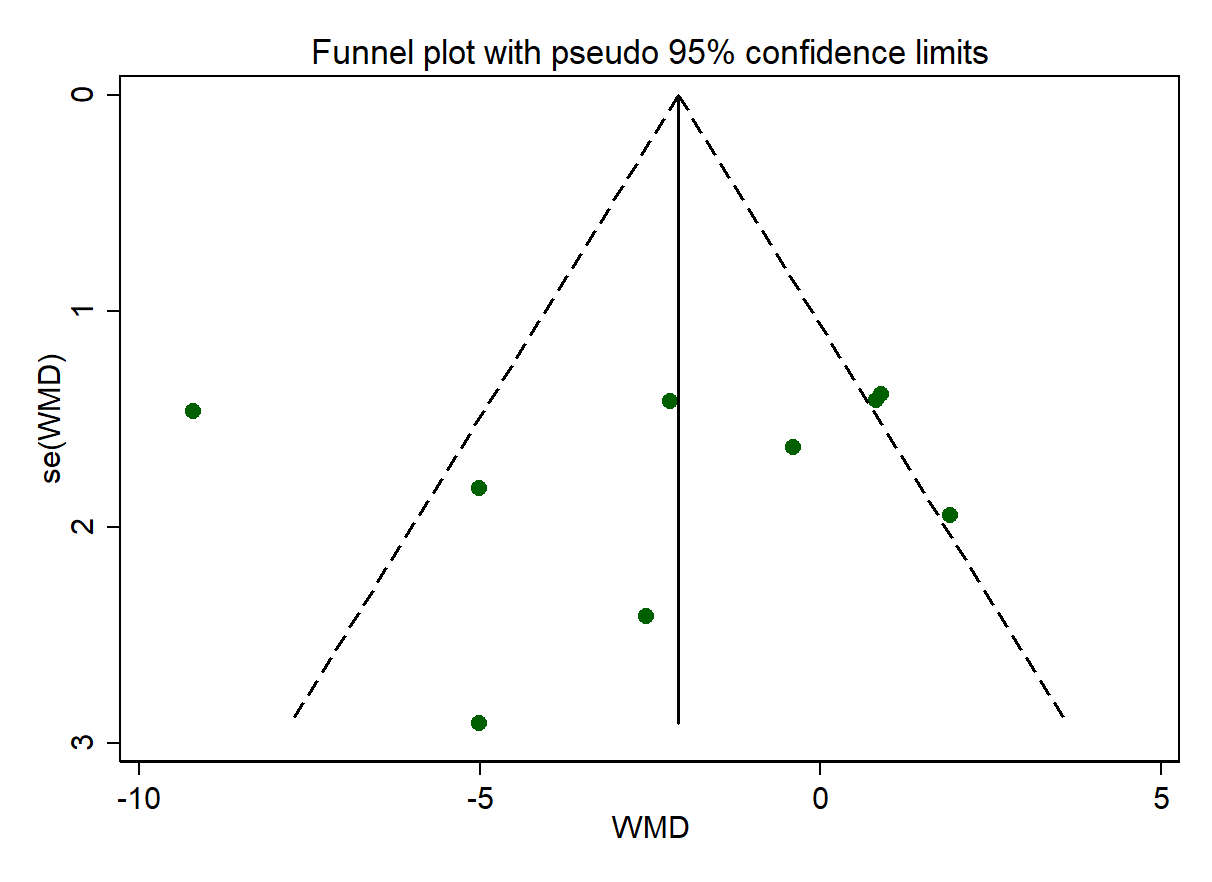

Supplement: Supplementary file 7 — Additional file 7: Figure-S7. Funnel plot of LVEF between thalassemic groups. [file 12947_2022_291_MOESM7_ESM.tif]

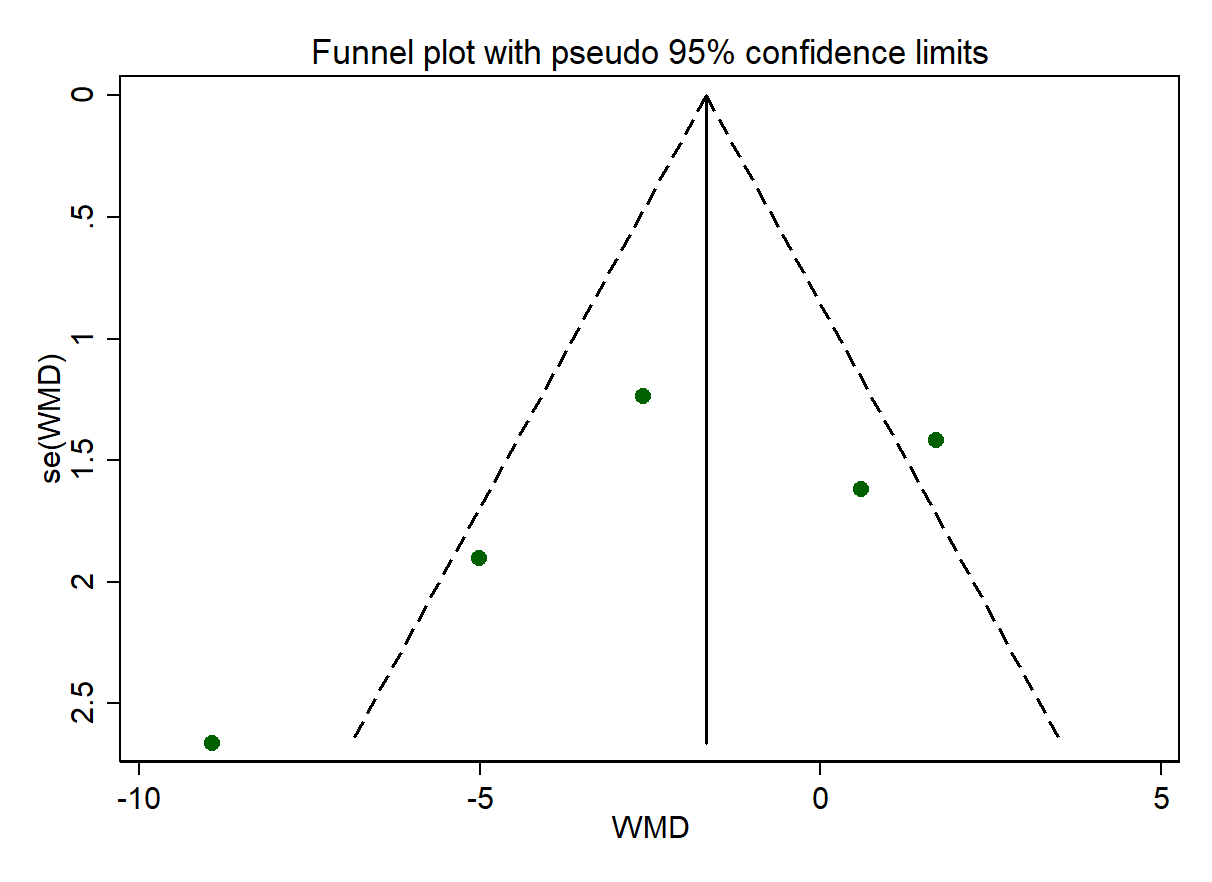

Supplement: Supplementary file 8 — Additional file 8: Figure-S8. Funnel plot of LVEF between thalassemic groups with CIO and healthy participants. [file 12947_2022_291_MOESM8_ESM.tif]
